# Characteristics of diffuse retinal nerve fiber layer defects in red-free photographs as observed in optical coherence tomography en face images

**DOI:** 10.1186/s12886-019-1302-z

**Published:** 2020-01-08

**Authors:** Abegaile Bartolome Lim, Ji-Hye Park, Jae Hoon Jung, Chungkwon Yoo, Yong Yeon Kim

**Affiliations:** 1 Department of Ophthalmology, Korea University College of Medicine, Seoul, South Korea; 2 Department of Ophthalmology, Korea University Ansan Hospital, 123 Jeokgeum-ro, Danwon-gu, Ansan-si, Gyeonggi-do 15355 South Korea

**Keywords:** En face image, Diffuse RNFL defect, OCT angiography, Red-free fundus photograph, Localized RNFL defect

## Abstract

**Backgroud:**

To determine whether diffuse retinal nerve fiber layer (RNFL) defects, identified on red-free fundus photographs, could be identified on optical coherence tomography (OCT) en face structural images and to evaluate which factors are related to the different recognition patterns on en face images.

**Methods:**

This retrospective, cross-sectional study included open-angle glaucoma eyes with diffuse RNFL defects in the inferior hemifield, identified in red-free photographs. The corresponding OCT en face structural images were divided into 3 groups: (1) no defect, (2) localized defect, and (3) diffuse defect. We compared the demographic and clinical ocular characteristics among the groups.

**Results:**

A total of 209 eyes from 157 patients were included. The distribution of OCT en face images was: no defect, 25 eyes (11.96%); localized defect, 106 eyes (50.72%); diffuse defect, 78 eyes (37.32%). Logistic regression analysis revealed that eyes with greater mean deviation (*P* = 0.004) and thicker inferior RNFL (*P* = 0.008) would be included in the no defect and localized defect groups based on OCT en face images, rather than in the diffuse defect group.

**Conclusion:**

Around half of diffuse RNFL defects identified in the red-free photographs appeared as localized defects in OCT en face images. Mild glaucomatous damage was related to no defect and localized defect groups, classified based on the OCT en face images, in eyes with diffuse photographic RNFL defects. OCT en face images may be helpful in further assessing diffuse RNFL defects seen in red-free photographs in eyes with open-angle glaucoma.

## Background

Glaucoma shows morphological changes to the optic nerve head (ONH) and retinal nerve fiber layer (RNFL) by progressive loss of retinal ganglion cells [1]. Evaluation of glaucoma progression is essential to patient management because progression can lead to irreversible loss of visual function. The visual field (VF) test is a standard tool for detecting glaucoma progression [2]. However, some glaucomatous eyes demonstrates only structural changes to the ONH and/or RNFL, without VF changes [3, 4]. Therefore, structural assessment is also important to evaluate the progression of glaucoma.

Red-free fundus photography helps to detect glaucomatous RNFL defects [5], which can be appeared as a dark stripe or wedge-shaped localized defect in the peripapillary area, parallel to the normal retinal striation, or as a diffuse loss of this striation [4–6]. Both localized and diffuse loss can be an initial sign of glaucomatous damage [7]. However, fundus photography is limited in its clinical value when patients have media opacity or if the underlying retinal layers are thin. In addition, using red-free fundus photography alone, it is difficult to detect mild glaucoma with diffuse RNFL defect [8].

Recently, Hood et al. [9] reported that glaucomatous damage is better visualized using optical coherence tomography (OCT) en face images. In their study, en face image showed details of RNFL defects that were hard to see with traditional RNFL thickness analysis. In addition, Jung et al. [10] reported a high topographic correlation between red-free fundus photographs and en face structural images in terms of RNFL defect location. However, both these studies included mostly localized defects, and they did not evaluate whether OCT en face structural images can identify diffuse RNFL defects better than red-free fundus photographs. In the current study, we evaluated the pattern of diffuse RNFL defect, as determined by red-free fundus photography, using OCT en face structural images.

## Methods

The medical records of patients with open-angle glaucoma (OAG) who had visited the Department of Ophthalmology, Korea University Guro Hospital from June 2016 through December 2016 were reviewed. All patients had undergone red-free fundus photography and OCT angiography within 6-month interval. This study was approved by the Institutional Review Board of the Korea University Guro Hospital and was performed according to the tenets of the Declaration of Helsinki.

The inclusion criteria were as follows: (1) age older than 18 years, (2) open angle on static gonioscopy, (3) glaucomatous ONH changes and corresponding VF defects, (4) diffuse RNFL defects in the inferior hemifield identified under red-free fundus photography. Patients who had a history or evidence of secondary OAG were excluded from the study, as were those who had undergone refractive surgery. However, patients with systemic hypertension and diabetes mellitus were included in the study.

All subjects underwent a complete ophthalmic examination, which included best-corrected visual acuity (BCVA), refractive error, slit-lamp biomicroscopy, Goldmann applanation tonometry, gonioscopy, dilated 30-degree stereoscopic fundus photography and 50-degree red-free photography using a FF 450 plus IR camera (Carl Zeiss Meditec Inc., Dublin, CA), OCT and OCT angiography (OCTA, Cirrus HD-OCT Model 5000 with AngioplexTM, Carl Zeiss Meditec Inc.) measurements, a Humphrey visual field test with the 24–2 Swedish Interactive Thresholding Algorithm (Zeiss Humphrey, San Leandro, CA), central corneal thickness, and axial length using an IOLMaster device (Carl Zeiss Meditec, Jena, Germany).

### Evaluation of glaucomatous RNFL defects in red-free fundus photographs

The red-free fundus photograph of each eye was independently analyzed in a random order by two blinded glaucoma specialists (A.B.L. and J.H.P.). Only cases with diffuse RNFL defect in the inferior hemifield, as indicated by red-free fundus photographs, were enrolled. Diffuse RNFL defect was identified when there was (1) loss of normal RNFL visibility or (2) a non-wedge defect with ill-defined proximal or distal borders [11]. For patients to be enrolled in the study, the two observers (A.B.L. and J.H.P.) had to agree that diffuse RNFL defect was present. In cases of disagreement, the opinion of a third observer (Y.Y.K.) was sought.

### Evaluation of glaucomatous RNFL defects in OCT en face images

Spectral domain OCT with OCT angiography (Cirrus HD-OCT Model 5000 with Angioplex™; Carl Zeiss Meditec Inc., Dublin, CA) was used to capture the OCT en face images. This OCT device offers a scan rate of 68,000 A-scans per second and operates a superluminescent diode with a center wavelength of 840 nm and a bandwidth of 90 nm. When en face flow images are obtained using OCT angiography, en face structural images can be achieved at the same time, and we used the latter in the analysis. The software of the OCT device offers automated segmentation of full-thickness retinal scans, and we used the superficial retinal layer segment (internal limiting membrane to inner plexiform layer). The images were evaluated by two observers (A.B.L. and J.H.P.), and images of poor quality, as determined by the following parameters, were excluded: (1) a signal strength index of <6 (minimum = 1, maximum = 10); (2) poor clarity; (3) motion artifacts, visible as irregular vessel patterns at the disc boundary on the en face structural images; (4) localized weak signal; and (5) segmentation errors of the superficial and whole retinal area.

RNFL defects on the OCT en face structural images (6 × 6 mm^2^; centered on the optic disc) were classified into three groups according to localization and shape of the RNFL defect: (1) no defect group; normal RNFL striations, (2) localized defect group; well-defined proximal and distal defect borders, the defect could be single or multiple, (3) diffuse defect group; loss of normal RNFL visibility or non-wedge defect with ill-defined proximal or distal borders [11].

### Optical coherence tomography imaging

The optic disc cube scan and ganglion cell analysis protocol for macular cube scanning were used to measure the thicknesses of circumpapillary RNFL and the ganglion cell inner plexiform layer (GCIPL), respectively. A circle scan 3.46 mm in diameter, consisting of 256 A-scans, was automatically positioned around the optic disc, and the average, 12 clock-hour, and 4-quadrant circumpapillary RNFL thicknesses were analyzed. The macular cube scan (macular cube: 512 × 128), centered on the 6 × 6-mm^2^ macular region, was performed and the following GCIPL thickness measurements were analyzed: average, minimum, and 6 sectoral values (superonasal, superior, superotemporal, inferotemporal, inferior, and inferonasal).

### Statistical analysis

Statistical analysis was performed using SPSS software version 21.0 (SPSS Inc., Chicago, IL). The Kruskal-Wallis test was performed to compare continuous variables among the three groups. To analyze the categorical data, Pearson’s chi-square test was performed. To compare differences between two groups, the Mann-Whitney U test was used. Univariate and multivariate logistic regression analyses were performed to determine which demographic and ocular characteristics could reliably predict inclusion in the no defect and localized defect groups. Variables with a *P* value less than 0.20 in the univariate analysis were entered into the multivariate analysis. All *P* values less than 0.05 were considered statistically significant.

## Results

A total of 209 eyes (104 right, 105 left) from 157 patients were included in this study. The mean age of the study participants was 57.69 ± 13.69 years (range: 27–84 years). The demographic and ocular characteristics of the participants are summarized in Table 1. The 209 eyes with diffuse defects of the inferior hemifield in red-free photography were categorized into one of the three groups based on the defect seen in the en face images: no defect, localized defect, or diffuse defect. The distribution was as follows: 25 eyes (11.96%) in the no defect group, 106 eyes (50.72%) in the localized defect group, and 78 eyes (37.32%) in the diffuse defect group. No significant differences were observed in terms of sex, eye laterality, intraocular pressure, central corneal thickness, and axial length among the three groups. However, the age, spherical equivalent (SE), mean deviation (MD), pattern standard deviation (PSD), and visual field index (VFI) differed significantly.

**Table 1:** Demographics of open-angle glaucoma patients

	No defect (*n* = 25)	Localized defect (*n* = 106)	Diffuse defect (*n* = 78)	*P* value*
Age (yrs)	63.96 ± 13.78	56.99 ± 13.31	56.63 ± 13.82	0.029
Number of female patients (%)	12 (48.0)	44 (41.5)	26 (33.3)	0.337^†^
Laterality (Right: Left)	10:15	57:49	37:41	0.406^†^
Diagnosis (%)				0.079^†^
NTG	15 (60.0)	45 (42.5)	27 (34.6)	
POAG	10 (40.0)	61 (57.5)	51 (65.4)	
Baseline IOP (mmHg)	16.1 ± 4.1	16.5 ± 3.6	18.7 ± 8.6	0.386
IOP (mmHg)	13.1 ± 3.1	13.6 ± 2.4	13.8 ± 3.0	0.674
SE (diopters)	−0.26 ± 3.41	−2.48 ± 4.41	− 2.16 ± 3.66	<0.001
CCT (μm)	538.61 ± 54.25	537.22 ± 35.77	532.50 ± 34.10	0.508
AXL (mm)	24.60 ± 2.11	25.11 ± 1.83	24.99 ± 2.19	0.492
MD (dB)	−4.50 ± 5.88	− 7.52 ± 6.72	−13.21 ± 8.74	<0.001
PSD (dB)	3.44 ± 2.22	7.41 ± 4.24	9.06 ± 4.50	<0.001
VFI (%)	92.31 ± 6.97	77.52 ± 21 .51	61.83 ± 24.72	<0.001

*Kruskal-Wallis Test, ^†^Pearson’s chi-square test

*NTG* Normal-tension glaucoma, *POAG* Primary open-angle glaucoma, *IOP* Intraocular pressure, *SE* Spherical equivalent, *CCT* Central corneal thickness, *AXL* Axial length, *MD* Mean deviation, *PSD* Pattern standard deviation

The Mann–Whitney U test was used to measure the differences in age, SE, MD, PSD, and VFI in a pairwise fashion among the three groups. In all five variables, a significant difference was observed between the groups (no defect group vs. localized defect group; no defect group vs. diffuse defect group, and localized defect group vs. diffuse defect group), except for age (*P* = 0.978) and SE (*P* = 0.639) when compared between the localized defect and diffuse defect groups.

Table 2 summarizes the RNFL thickness measurements in the three groups. Significant differences were found in average RNFL thickness, RNFL quadrant thickness, and RNFL clock hour thickness, other than at the 3 o’clock (*P* = 0.891) and 9 o’clock (*P* = 0.092) positions. Since only RNFL defects in the inferior hemifield were included in the present study, we focused on the inferior RNFL quadrant thickness and at the 6 to 9 o’clock positions. Comparing the aforementioned RNFL thicknesses in a pairwise fashion among the groups, there was a significant difference in terms of average RNFL thickness, inferior RNFL thickness, and thickness at the 6 to 9 o’clock positions, except between the localized defect and diffuse defect groups at the 7 o’clock (*P* = 0.187) and 8 o’clock (*P* = 0.100) positions. At the 9 o’clock position, no significant differences were noted between the no defect and localized defect groups (*P* = 0.367) or between the no defect and diffuse defect groups (*P* = 0.389).

**Table 2:** Comparison of retinal nerve fiber layer thickness (μm) among the three groups

	No defect (*n* = 25)	Localized defect (*n* = 106)	Diffuse defect (*n* = 78)	*P* value*
Average	79.52 ± 13.30	69.51 ± 10.00	63.46 ± 10.61	<0.001
Superior	92.48 ± 22.98	86.12 ± 20.14	76.06 ± 20.46	<0.001
Nasal	65.64 ± 10.27	62.87 ± 8.91	60.94 ± 10.34	0.033
Inferior	99.52 ± 19.68	70.40 ± 13.65	62.03 ± 12.72	<0.001
Temporal	60.60 ± 13.18	57.93 ± 11.52	53.91 ± 12.85	0.011
1 o’clock	94.64 ± 25.57	82.92 ± 20.53	74.94 ± 24.03	<0.001
2 o’clock	74.44 ± 14.84	70.80 ± 11.72	67.90 ± 14.24	0.048
3 o’clock	58.16 ± 9.65	59.08 ± 10.69	58.44 ± 11.00	0.891
4 o’clock	64.04 ± 10.20	58.58 ± 9.32	56.74 ± 9.81	0.003
5 o’clock	82.60 ± 16.60	73.35 ± 15.50	62.54 ± 12.97	<0.001
6 o’clock	109.24 ± 23.91	72.75 ± 2 1.47	60.50 ± 16.85	<0.001
7 o’clock	106.96 ± 28.45	65.55 ± 17.47	62.88 ± 18.05	<0.001
8 o’clock	63.76 ± 16.71	55.04 ± 13.22	51.90 ± 12.62	0.002
9 o’clock	51.36 ± 10.24	53.05 ± 11.88	51.00 ± 16.68	0.092
10 o’clock	66.76 ± 15.44	67.79 ± 17.20	58.85 ± 17.12	0.012
11 o’clock	88.72 ± 27.30	89.24 ± 28.54	77.90 ± 25.62	0.021
12 o’clock	94.92 ± 31.26	88.21 ± 24.80	74.85 ± 26.15	<0.001

*Kruskal-Wallis Test

Table 3 shows the GCIPL thickness and disc parameters among the three groups. Average GCIPL thickness, minimum GCIPL thickness, all six GCIPL macular sections, and the disc parameters showed significant differences among the three groups. However, no significant differences occurred in average GCIPL (*P* = 0.264), minimum GCIPL (*P* = 0.071), and inferonasal GCIPL thickness (*P* = 0.246) between the no defect and localized defect groups. With regards to disc parameter, when cup volume was compared using the Mann–Whitney U test, no significant difference was found between the no defect and localized defect groups (*P* = 0.949).

**Table 3:** Comparison of ganglion cell-inner plexiform layer (GCIPL) thickness and disc parameters among the three groups

	No defect (*n* = 25)	Localized defect (*n* = 106)	Diffuse defect (*n* = 78)	*P* value*
GCIPL thickness (μm)				
Average	67.48 ± 12.20	65.80 ± 8.46	61.42 ± 8.89	<0.001
Minimum	60.08 ± 16.67	54.75 ± 11.23	50.87 ± 11.30	0.006
Superior	66.64 ± 13.26	70.32 ± 40.55	64.26 ± 11.54	0.001
Superonasal	71.16 ± 11.18	73.20 ± 11.04	67.82 ± 13.57	0.004
Inferonasal	69.72 ± 10.49	67.06 ± 10.42	61.56 ± 10.06	<0.001
Inferior	68.40 ± 10.60	59.04 ± 9.78	56.47 ± 9.34	<0.001
Inferotemporal	70.24 ± 11.38	57.83 ± 8.71	55.26 ± 9.92	<0.001
Superonasal	66.40 ± 11.59	10.32 ± 1.00	12.19 ± 1.38	0.009
Disc parameters				
Rim area (mm^2^)	1.02 ± 0.24	0.77 ± 0.18	0.67 ± 0.19	<0.001
Disc area (mm^2^)	2.34 ± 0.47	2.02 ± 0.48	2.12 ± 0.50	0.018
Average cup to disc ratio	0.73 ± 0.09	0.77 ± 0.08	0.81 ± 0.07	<0.001
Vertical cup to disc ratio	0.72 ± 0.09	0.78 ± 0.09	0.81 ± 0.08	<0.001
Cup volume (mm^3^)	0.49 ± 0.28	0.50 ± 0.29	0.63 ± 0.31	0.010

* Kruskal-Wallis Test

Univariate and multivariate logistic regression analyses were performed with the dependent variable being included into either the no defect or localized defect groups (Table 4). Univariate analysis showed that MD, average RNFL thickness, inferior RNFL thickness, average GCIPL thickness, and minimum GCIPL thickness differed significantly between the groups (*P* < 0.05). However, in multivariate analysis, MD (Exp [B] = 1.077; *P* = 0.004) and inferior RNFL thickness (Exp [B] = 1.047; *P* = 0.008) independently predicted inclusion into either the no defect or localized defect group.

**Table 4:** Logistic regression analysis with the dependent variable being included either into no defect or into localized defect groups

	Univariable			Multivariable		
	Exp (B)	95% CI	*P* value	Exp (B)	95% CI	*P* value
Age	1.009	0.989–1.030	0.387			
Gender (Female)	0.670	0.373–1.201	0.179	0.777	0.394–1.534	0.467
Laterality (left eye)	1.160	0.662–2.033	0.604			
SE	1.009	0.932–1.091	0.837			
AXL	1.008	0.853–1.184	0.953			
CCT	1.004	0.996–1.011	0.364			
Diagnosis (NTG)	1.596	0.894–2.849	0.114	0.969	0.480–1.954	0.929
MD	1.108	1.065–1.153	<0.001	1.077	1.024–1.132	0.004
Average RNFL thickness	1.071	1.039–1.103	<0.001	1.003	0.952–1.057	0.909
Inferior RNFL thickness	1.064	1.038–1.090	<0.001	1.047	1.012–1.084	0.008
Average GCIPL thickness	1.059	1.025–1.095	0.001	0.988	0.921–1.060	0.742
Minimum GCIPL thickness	1.035	1.010–1.061	0.006	0.992	0.947–1.040	0.752

*CI* Confidence interval, *SE* Spherical equivalent, *AXL* Axial length, *CCT* Central corneal thickness, *NTG* Normal-tension glaucoma, *MD* Mean deviation, *RNFL* Retinal nerve fiber layer, *GCIPL* Ganglion cell inner plexiform layer

## Discussion

**Fig. 1:**
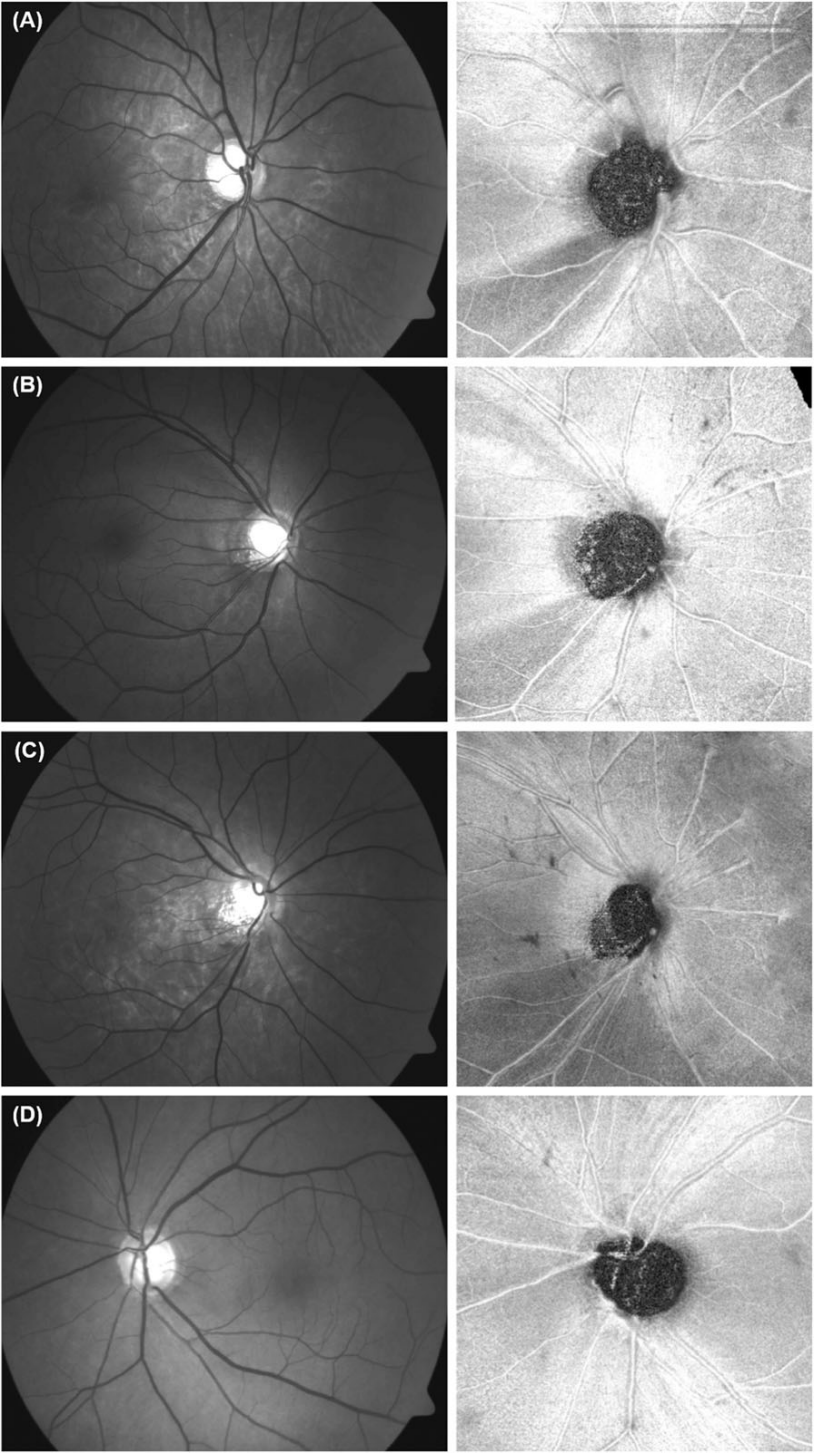
Representative cases of differences in detecting diffuse retinal nerve fiber layer (RNFL) defect between the red-free fundus photographs (left panel) and en face structural images (right panel). **a**, **b** The red-free fundus photographs show a diffuse RNFL defects, while the en face images clearly show localized RNFL defects. **c** The image shows a diffuse RNFL defect in the inferior hemifield, both in the red-free fundus photograph and the en face image. The peripheral borders of the defect in the en face image are indistinct. **d** The red-free photo shows a diffuse RNFL defect in the inferior quadrant; however, the en face image shows no RNFL defect

In the present study, we evaluated whether diffuse RNFL defect, as seen on red-free fundus photography, could be discerned on OCT en face structural images. After evaluating the latter, not all lesions classified as diffuse RNFL defects based on red-free photographs had been correctly identified in the en face image. Instead, some defects showed different patterns in the en face images. Specifically, out of the 209 eyes included in this study, which had been diagnosed as having diffuse defects based on the red-free photographs, 50.72% appeared as localized defects in the en face images (Fig. 1a and b), while 37.32% were diffuse defects (Fig. 1c), and 11.96% had no defects at all (Fig. 1d). To our knowledge, no studies have described the patterns of diffuse RNFL defects in en face images, although studies on localized defects in en face images have been conducted [9, 10].

Red-free fundus photography is the standard method of assessing glaucomatous damage of the RNFL or of determining the progression of RNFL defects. In this technique, a green filter (540–570 nm) is used to block out red wavelengths of light and allow better contrast for viewing subtle changes in the RNFL. However, fundus photography has its clinical limitations because interpretation of the images is qualitative and subjective. To better assess subtle RNFL changes and quantitatively measure RNFL thickness, OCT is commonly used in the clinic. Several studies have reported that red-free fundus photography and OCT maps agree in terms of identifying the RNFL defect [12–15]. Hwang et al. [13] reported that red-free fundus photographs agreed well with Cirrus HD-OCT RNFL thickness maps in terms of the angular locations of RNFL defect margins in glaucomatous eyes. In addition, when the diagnostic abilities of clock-hour, deviation, and thickness maps compiled using Cirrus HD-OCT were compared, the thickness map was the most effective in detecting photographic RNFL defects [14].

Although OCT thickness maps are useful, OCT en face images may show more details of the RNFL abnormalities that are difficult to see using previously available methods. As Hood et al. [9] suggested, OCT en face images are superior to the OCT thickness map for several reasons. Firstly, since the abnormal regions of the RNFL vary in thickness and reflectance intensity, en face images may capture both abnormalities unlike RNFL thickness maps. Secondly, blood vessels can be confused for local RNFL bundle preservation on thickness maps, while they are easier to distinguish on en face images. Similarly, subtle arcuate RNFL defects, which are not obvious on RNFL thickness maps, can be identified easily on en face images.

In the present study, the details of the diffuse RNFL defects were observed in the OCT en face images. The defects were more hyper-reflective in these images, and their peripheral borders, whether distinct or indistinct, were better visualized. Moreover, in cases of localized defect and multiple defects, normal areas of the RNFL were clearly observed in the en face images. As expected, the differing patterns of the OCT en face images correlated with a decreasing trend in MD, VFI, RNFL thickness, GCIPL thickness, and rim area. Lesions identified as diffuse defects in red-free fundus photography may have been recognized as normal or as localized defect in the en face images for several reasons. Firstly, diffuse defects could be missed if the local RNFL thickness was not reduced to less than the thickness of the segment used in the en face image. In fact, the MD and inferior RNFL thickness were independently associated with inclusion into the no defect or localized defect group, rather than in the diffuse defect group. Specifically, when the MD value increased by 1 dB, there was a 1.077- × higher chance that the patient would be assigned to the no defect or localized defect group, and when the inferior RNFL thickness value increased by 1 μm, there was a 1.047- × higher chance that the patient would be classified into one of these groups. Secondly, the reflectance intensity of the RNFL, which declines secondary to glaucomatous damage, may have not been reduced enough to be captured in the en face image. Therefore, although the en face image provides more details regarding the RNFL, we cannot conclude that red-free fundus photography can be completely replaced by analysis of reflectance intensity in OCT en face images to detect diffuse RNFL defect. Instead, information from en face images can supplement red-free fundus images and/or OCT RNFL thickness maps.

The present study has several limitations. Firstly, we did not compare the angular locations of the RNFL defects between the red-free fundus photographs and the en face images. Secondly, the patterns of VF defect were not analyzed, even though the patients had glaucomatous VF defects that corresponded to the ONH changes. Thirdly, only eyes with diffuse RNFL defects in red-free fundus photography were included. Therefore, further studies are necessary to compare localized RNFL abnormalities, as determined using red-free fundus photography, with en face structural images. Furthermore, although two glaucoma specialists independently determined the presence of diffuse RNFL defect in red-free fundus photography, the recognition of RNFL defect in red-free fundus photograph could be somewhat subjective.

## Conclusions

In conclusion, using the OCT en face imaging in conjunction with standard red-free fundus photographs can be helpful in determining and assessing RNFL defects, especially in glaucomatous eyes with diffuse RNFL defects.

## Data Availability

The datasets used and/or analysed during the current study are available from the corresponding author on reasonable request.

## Abbreviations

AXL: Axial length
BCVA: Best-corrected visual acuity
CCT: Central corneal thickness
GCIPL: Ganglion cell inner plexiform layer
IOP: Intraocular pressure
MD: Mean deviation
OAG: Open-angle glaucoma
OCT: Optical coherence tomography
OCTA: Optical coherence tomography angiography
ONH: Optic nerve head
PSD: Pattern standard deviation
RNFL: Retinal nerve fiber layer
SE: Spherical equivalent
VF: Visual field
VFI: Visual field index
